# Immunogenicity study of a *Streptococcus suis* autogenous vaccine in preparturient sows and evaluation of passive maternal immunity in piglets

**DOI:** 10.1186/s12917-021-02774-4

**Published:** 2021-02-05

**Authors:** Lorelei Corsaut, Léa Martelet, Guillaume Goyette-Desjardins, Guy Beauchamp, Martine Denicourt, Marcelo Gottschalk, Mariela Segura

**Affiliations:** 1 Research Group on Infectious Diseases in Production Animals (GREMIP) and Swine and Poultry Infectious Diseases Research Centre (CRIPA), Faculty of Veterinary Medicine, University of Montreal, 3200 Sicotte St., Saint-Hyacinthe, Quebec, J2S 2M2 Canada; 2 Biostatistics Office, Faculty of Veterinary Medicine, University of Montreal, Saint-Hyacinthe, Quebec, Canada

**Keywords:** *Streptococcus suis*, Infection, Field, Autogenous bacterin, Vaccine, Pigs

## Abstract

**Background:**

*Streptococcus suis* is an important pathogen that causes severe diseases mostly in weaned piglets. Only available vaccines in the field are those composed of killed bacteria (bacterins) but data about their effectiveness are missing. We report here a field study on the immunological response induced by an autogenous vaccine applied in pre-parturient sows. Using a farm with recurrent *S. suis* serotype 7 problems, the study was divided in three experiments: (I) Sows received the vaccine at 7 and 3 weeks pre-farrowing. (II) Replacement gilts introduced to the herd received the vaccine at 4 and 7 weeks after their entry in quarantine and a boost 3 weeks pre-farrowing. (III) Gilts from experiment II received another boost 3 weeks pre-farrowing at their 3rd/4th parity. Levels, isotype profile and opsonophagocytosis capacity of the serum antibodies induced by vaccination were evaluated in sows and maternal immunity in piglets.

**Results:**

In sows (I), the vaccine induced a slight, albeit significant, increase in anti-*S. suis* total antibodies after 2 doses when compare to basal levels already present in the animals. These antibodies showed a high opsonic capacity in vitro, highlighting their potential protective capacity. A gilt vaccination program of 3 doses (II) resulted in a significant increase in anti-*S. suis* total antibodies. Levels of maternal immunity transferred to piglets were high at 7 days of age, but rapidly decreased by 18 days of age. A gilt vaccination program ensued a higher transfer of maternal immunity in piglets compared to control animals; nevertheless duration was not improved at 18 day-old piglets. The vaccine response in both gilts and sows was mainly composed of IgG1 subclass, which was also the main Ig transferred to piglets. IgG2 subclass was also found in piglets, but its level was not increased by vaccination. Finally, a recall IgG1 response was induced by another boost vaccination at 3rd/4th parity (III), indicating that the vaccine induced the establishment of a lasting memory response in the herd.

**Conclusions:**

Overall, an optimal gilt/sow vaccination program might result in increased antibody responses; nevertheless duration of maternal immunity would not last long enough to protect post-weaned piglets.

**Supplementary Information:**

The online version contains supplementary material available at 10.1186/s12917-021-02774-4.

## Background

*Streptococcus suis* is an encapsulated bacterium which causes numerous pathologies, such as meningitis, arthritis, endocarditis, polyserositis and septicemia with sudden death. It is responsible for important economic losses in the swine industry [1]. Formerly, 35 serotypes have been reported. However, recent taxonomical studies suggested that the reference strains of serotypes 20, 22, 26, 32, 33 and 34 should not be included within the *S. suis* species [2]. The repartition of serotypes that cause disease in pigs can vary worldwide. While in Europe serotypes 2 and 9 are the most frequently isolated from clinical cases [3], the situation in North America is more complex with a large number of serotypes frequently isolated from diseased animals. In addition, *S. suis* has been reported to be an emerging zoonotic pathogen with the greatest risk for people who have close contact with pigs or unprocessed pork [4].

The natural habitat of *S. suis* is the upper respiratory tract of pigs, and the transmission of this pathogen among animals occurs from sows to piglets and between piglets. Pigs are affected generally between 5 and 10 weeks of age, when levels of passive maternal immunity have decreased [1, 5]. Presently, autogenous bacterins are the only type of vaccines used in the field to prevent *S. suis* disease; however, this approach has resulted in contradictory results in terms of protective capacity and its application remains empirical [6, 7]. In the field, autogenous bacterins are applied to preparturient gilts/sows, piglets or, exceptionally, to both [8]. Though, the maternal immunity interference can be a problem for the vaccination of piglets [9]. Indeed, some studies have demonstrated that neither vaccination of suckling nor of weaned piglets from immunized sows was associated with a prominent active immune response and protection, explained by a potential inhibitory effect of maternal antibodies [5, 6]. Immunization of preparturient sows might elicit protective passive maternal immunity to their progeny and can be an attracting alternative to piglet vaccination [9]. However, results from vaccinated sows with bacterins are also matter of controversy [5, 9–11].

Independently of the vaccination program (sow vs. piglets), the limited protective response generally observed with bacterins can be explained by many hypotheses. For example, fixation with formalin or heat treatment might degrade bacterial epitopes and consequently vaccine immunogenicity [11, 12]. Bacterial concentration and number of vaccine doses would also be a limiting factor, with repeated immunizations probably required to induce protection [12].

In a recent critical review, Rieckmann et al. [11] reported that an important disadvantage of autogenous vaccines is the lack of information on vaccine efficacy and also the scarce information on the immunogenicity and/or protective efficacies of vaccines containing other serotypes than 2. To fill the knowledge gap on the immunological response induced by autogenous vaccines and transfer of immunity to piglets, herein, a field study was performed to evaluate a sow vaccination program using an autogenous bacterin in a herd with history of *S. suis* serotype 7 problems. *S. suis* serotype 7 is frequently isolated from clinical cases in pigs, especially in North America [3, 4]. A comparative analysis was performed in gilts and sows and the humoral response (levels, isotypes, and killing capacity of generated antibodies) was evaluated as well as the duration of the memory response induced by the vaccine. Finally, maternal immunity transfer to piglets was characterized, as this is the expected outcome of a sow vaccination program.

## Results

**Fig. 1:**
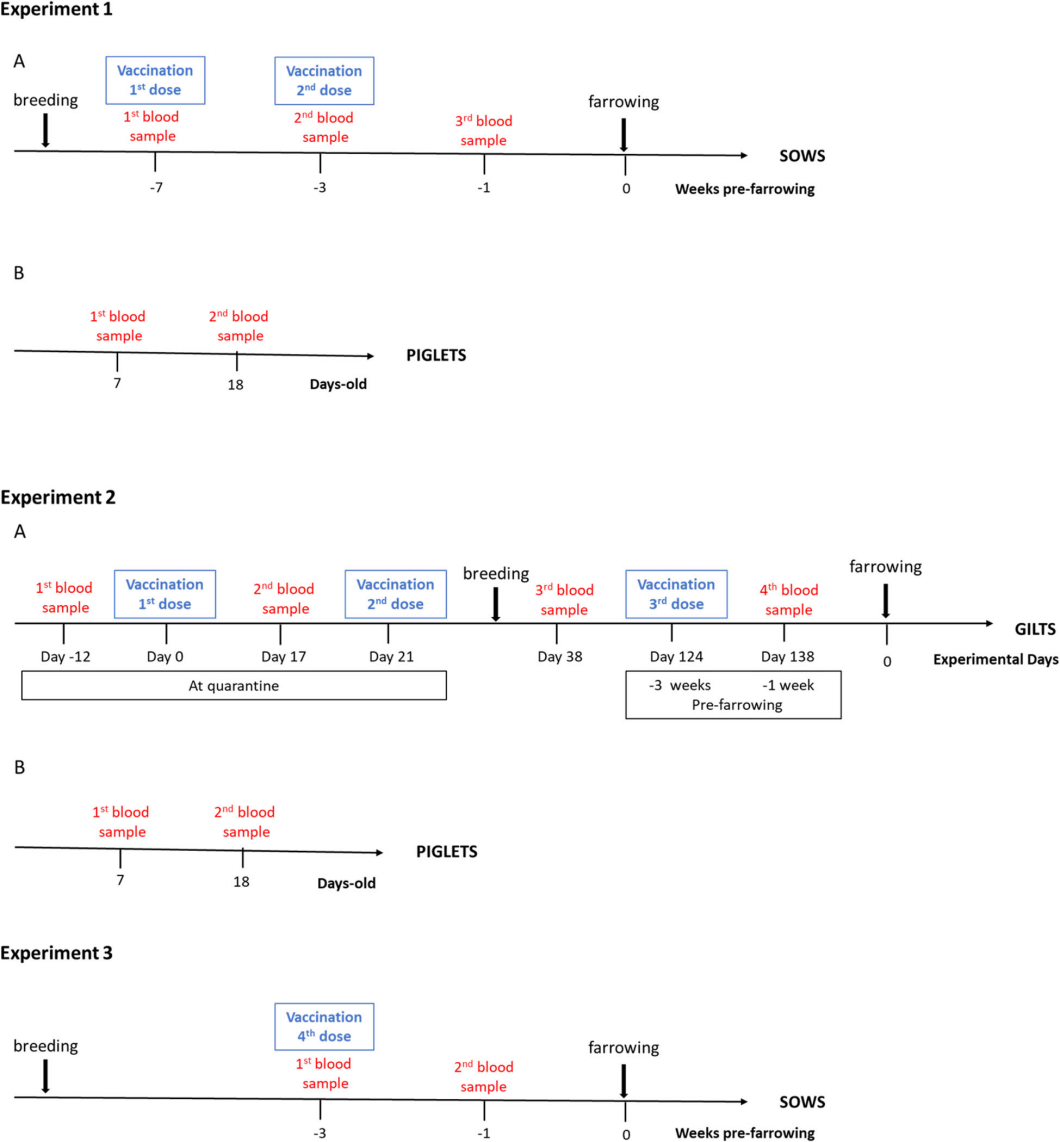
Experimental design of the field study. Experiment 1: (A) Sows received 2 doses of the autogenous vaccine intramuscularly at 7 and 3 weeks before farrowing. Blood samples were collected from all enrolled sows just prior to vaccination at 7 weeks before farrowing, at 5 weeks before farrowing (2 weeks after the 1st vaccination) and at 1 week before farrowing (2 weeks after the 2nd vaccination). (B) Randomly selected piglets from vaccinated sows were tagged, then sampled at 7 and 18 days of age. Experiment 2: (A) Gilts received 3 doses of the autogenous vaccine intramuscularly: during quarantine (at the indicated time points) and after quarantine (3 weeks before farrowing). Blood samples were collected from all enrolled gilts prior to vaccination and after each vaccine dose as indicated. (B) Randomly selected piglets from vaccinated and non-vaccinated gilts were tagged, then sampled at 7 and 18 days of age. Experiment 3: Previous gilts from experiment 2 received a single boost-dose (4th dose) of the autogenous vaccine intramuscularly at their 3rd or 4th parity according to their reproductive performance (3 weeks before farrowing). Blood samples were collected prior to vaccination and at 1 week before farrowing

Experimental design is presented in Fig. 1 and details are available in Methods’ section.

### Experiment 1: sow vaccination

#### Antibody levels induced by the autogenous vaccine increased in vaccinated sows, with an isotype response dominated by the IgG1 subclass

**Fig. 2:**
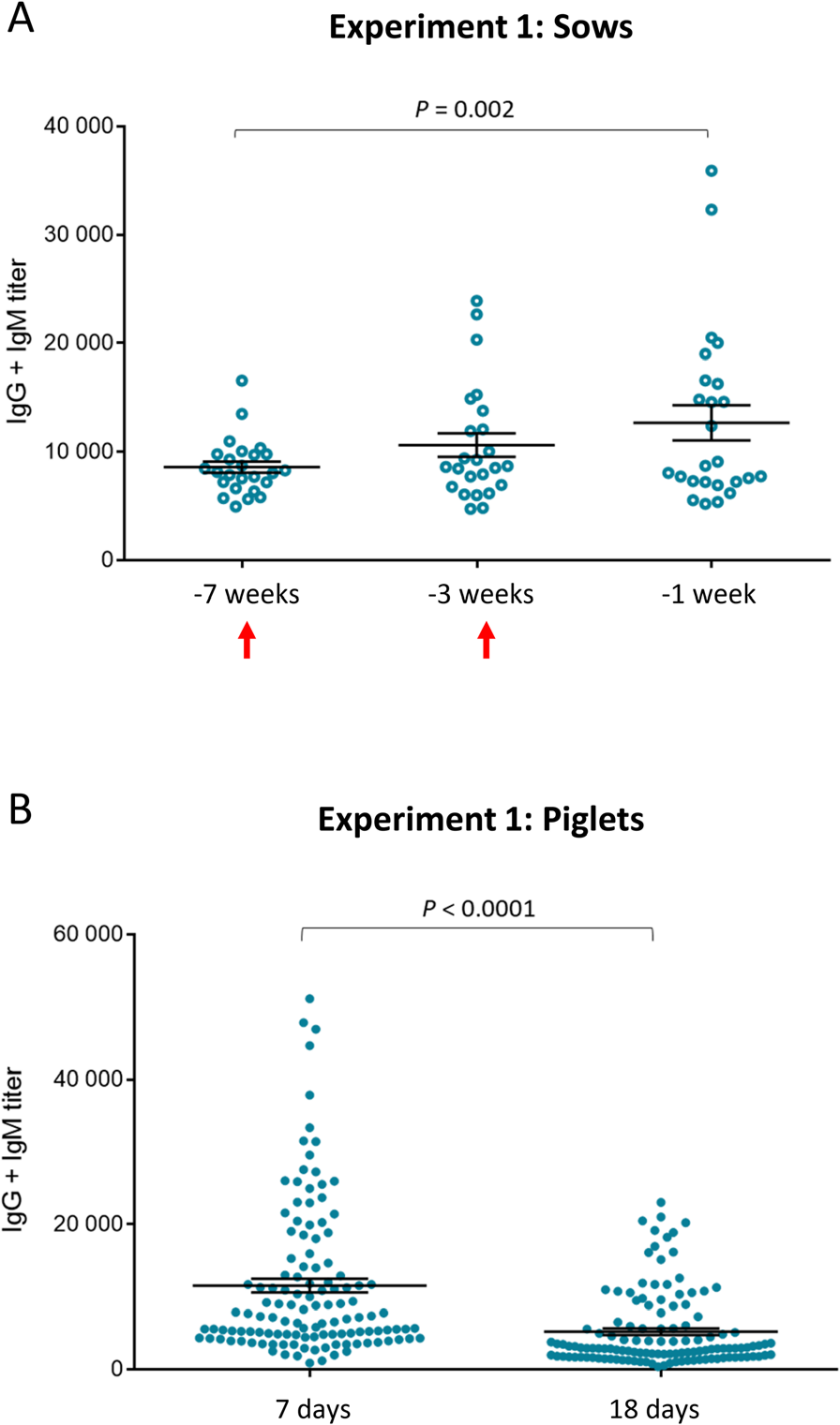
Experiment 1: Kinetics of total Ig against *S. suis* serotype 7 in vaccinated sows and their piglets. **a** Blood samples were collected at 7 weeks, 3 weeks and 1 week before farrowing from 25 vaccinated sows to follow the immune response. The vaccination protocol is shown in Fig. 1. Total Ig [IgG + IgM] titers were determined by ELISA. Arrows indicate 1st and 2nd vaccination doses. **b** Randomly selected piglets (*n* = 125; 5 piglets/sow) were sampled at 7 and 18 days of age and total Ig [IgG + IgM] titers were determined by ELISA against *S. suis* serotype 7. Individual antibody titers are shown with horizontal bars representing mean ± SEM. Values significantly different are shown in the graphs with corresponding *P* value

**Fig. 3:**
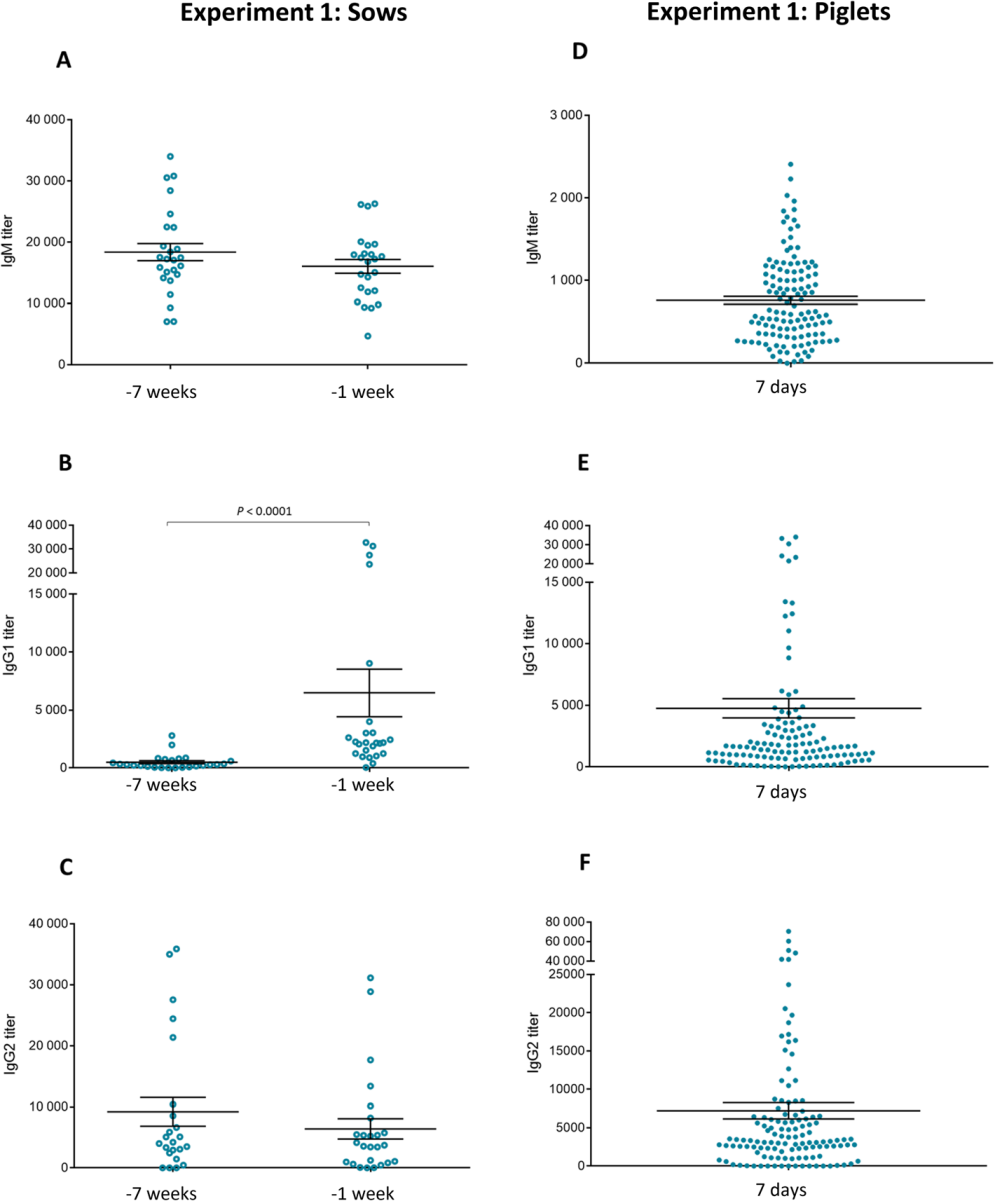
Experiment 1: Isotype profile of antibodies against *S. suis* serotype 7 in vaccinated sows and their piglets. Blood samples were collected 7 weeks and 1 week before farrowing from 25 vaccinated sows to analyze the immune response. The vaccination protocol is shown in Fig. 1. IgM (**a**), IgG1 (**b**) and IgG2 (**c**) titers were determined by ELISA. Randomly selected piglets (*n =* 125; 5 piglets/sow) from vaccinated sows were sampled at 7 days of age to evaluate titers of IgM (**d**) IgG1 (**e**) and IgG2 (**f**) against *S. suis* serotype 7. Individual antibody titers are shown with horizontal bars representing mean ± SEM. Values significantly different are shown in the graphs with corresponding *P* value

In Experiment 1, the antibody response induced by a 2-dose vaccination program in pre-parturient sows with an autogenous vaccine was evaluated (it should be noted that animals were never vaccinated against *S. suis* before). Unfortunately, the *S. suis* vaccination program, for all sows pre-farrowing of the herd, had just started when the study was initiated; therefore a control non-vaccinated group could not be included. As shown in Fig. 2a, a relatively high basal level of antibodies reacting against the *S. suis* serotype 7 vaccine strain was already present in sows. Primary immunization failed to significantly increase the titers of total Ig [IgG + IgM] against the vaccine strain compared to basal levels. A small, albeit significant, increase compared to basal levels was only observed after the second vaccine dose (Fig. 2a). When the isotype profile (IgM, IgG1 and IgG2) was analyzed, a significant increase of IgG1 (Fig. 3b) was observed after boost vaccination compared to basal levels; whereas levels of IgM and IgG2 remained equal before and after vaccination (Fig. 3a and c). Due to the lack of a control group, only a global characterization of maternal immunity in piglets was performed. At 7 days of age, piglets presented high levels of total Ig [IgG + IgM] against *S. suis* serotype 7 (Fig. 2b). This response was composed of IgG1, IgG2 and low levels of IgM (Figs. 3d-f). Antibodies levels in piglets dropped very fast by 18 days of age (Fig. 2b). Finally, a positive and statistically significant association was found between sow antibody titers and those observed in piglets (*P* < 0.0001).

#### Antibodies present in sows are highly opsonic and able to induce *S. suis* killing by blood leukocytes

**Fig. 4:**
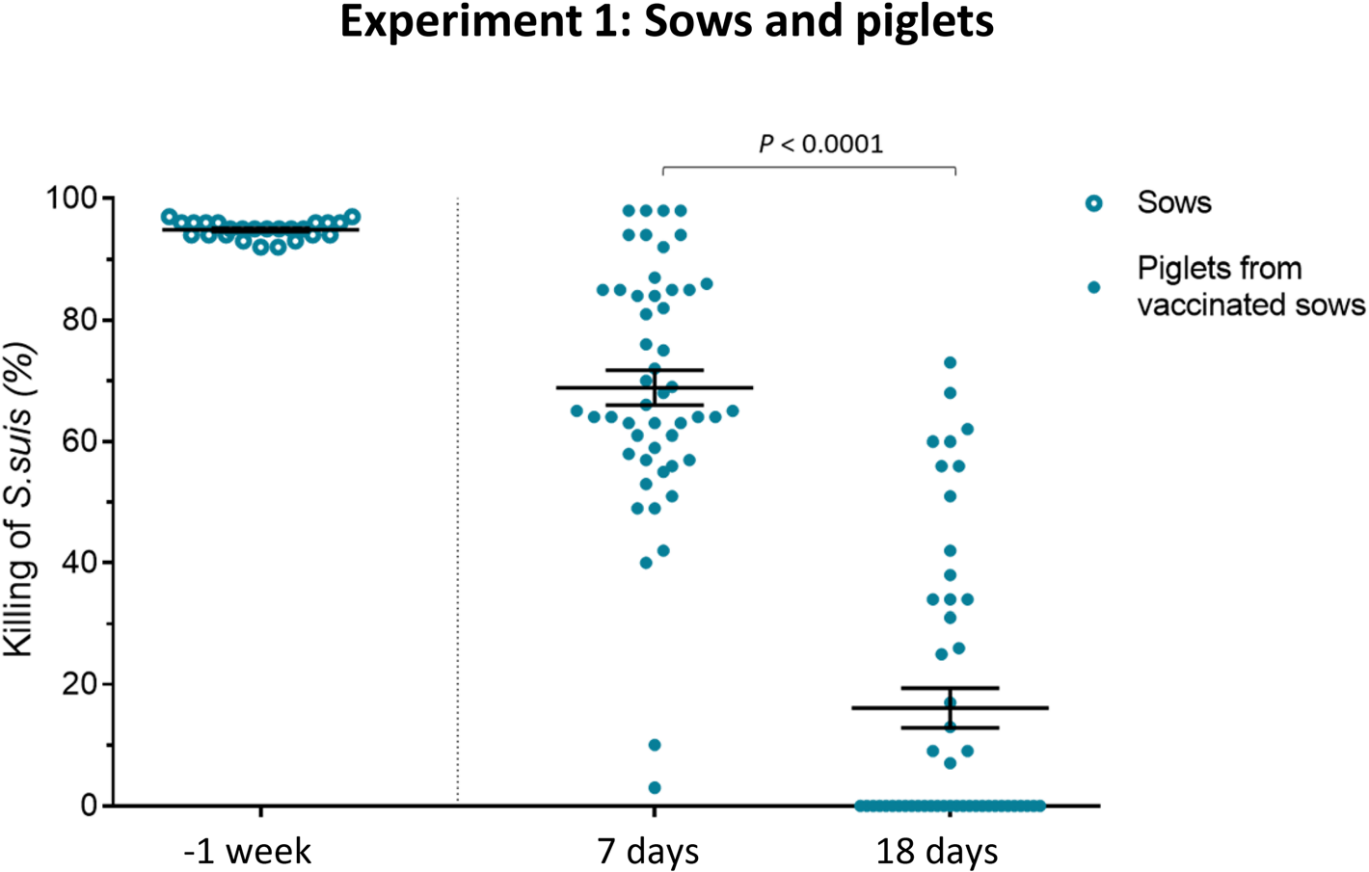
Experiment 1: Opsonophagocytosis killing of *S. suis* serotype 7 induced by serum antibodies from vaccinated sows and from their piglets. Blood samples were collected 1 week before farrowing from 25 vaccinated sows and from randomly chosen piglets (*n* = 50; 2 per sow) at 7 and 18 days of age to evaluate antibody functionality in an opsonophagocytosis assay (OPA). For OPA, blood leukocytes were mixed with *S. suis* serotype 7 (vaccine strain) at a multiplicity of infection of 0.01. Control sera or sample sera were added to a final concentration of 40% v/v in microtubes which were incubated for 4 h. After incubation, viable bacterial counts were performed, and the percentage of bacterial killing determined. Results are expressed as the % of *S. suis* killing for individual serum, with horizontal bars representing mean ± SEM. Values significantly different are shown in the graph with corresponding *P* value

Sera from sows 1 week before farrowing (after 2 vaccinations) and from their piglets at 7 and 18 days of age were evaluated in the opsonophagocytosis test (OPA). This test evaluates the capacity of vaccine-induced antibodies to kill bacteria in the presence of phagocytic cells. As shown in Fig. 4, the average OPA activity of antibodies in sows 1 week before farrowing (after 2 vaccinations) was about 95%. Due to maternal transfer of these functionally active antibodies, OPA capacity was also high in the sera of piglets from these vaccinated sows at 7 days, whose average was about 70% (Fig. 4). Yet, the OPA activity of these antibodies was significantly reduced at 18 days of age (*P* < 0.0001).

### Experiment 2: gilt vaccination

#### A 3-dose vaccination program of replacement gilts induced a rapid increase in antibody levels

**Fig. 5:**
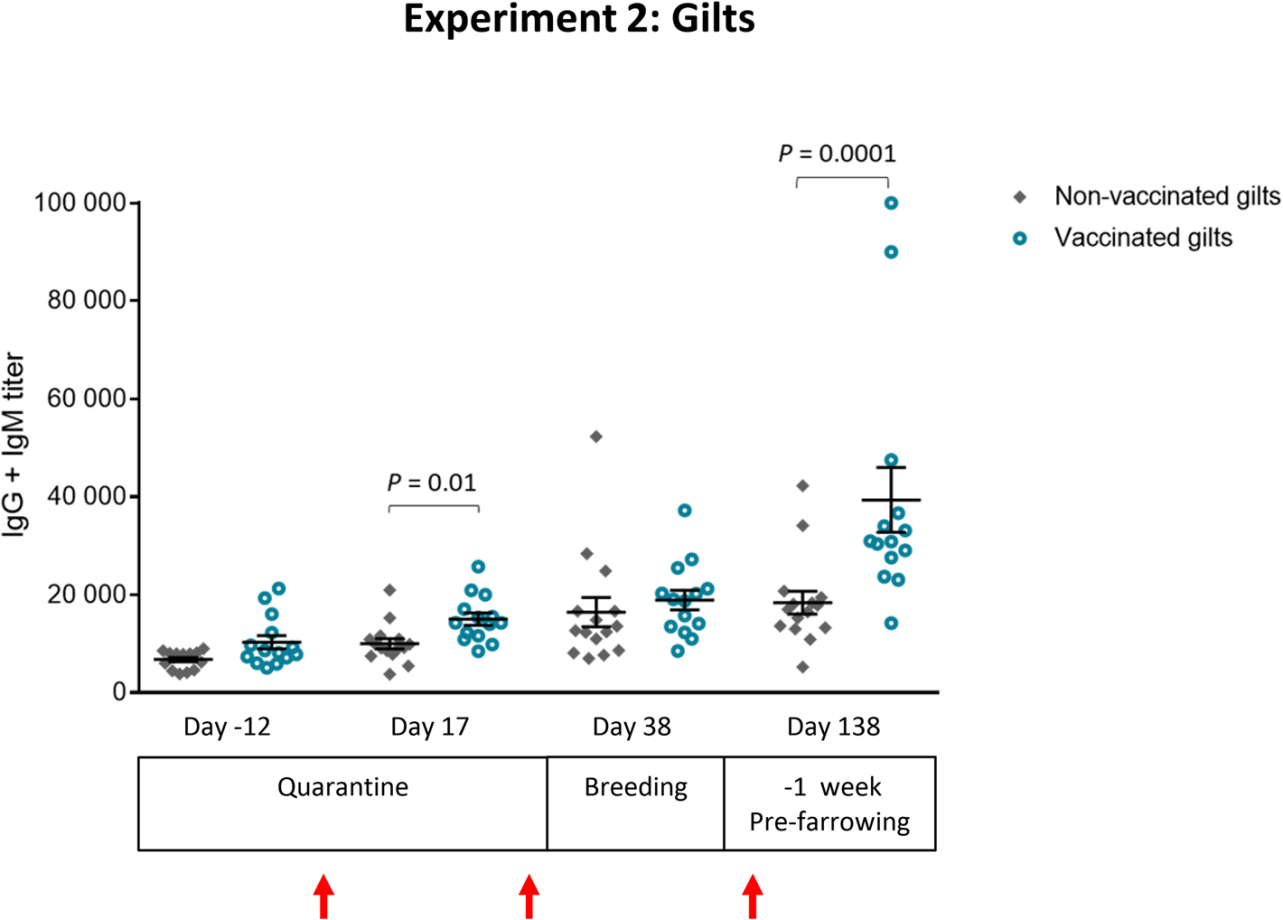
Experiment 2: Kinetics of total Ig against *S. suis* serotype 7 in replacement gilts. Blood samples were collected from 14 vaccinated and 15 non-vaccinated gilts prior to vaccination at day − 12, at day 17 (2 weeks after the 1st vaccination), at day 38 (2 weeks after the 2nd vaccination) and at day 138 (2 weeks after the 3rd vaccination). The vaccination protocol is shown in Fig. 1. Total Ig [IgG + IgM] titers were determined by ELISA. Individual antibody titers are shown with horizontal bars representing mean ± SEM. Values significantly different are shown in the graph with corresponding *P* value. Arrows indicate 1st, 2nd and 3rd vaccination doses

The second experiment assessed the immune response induced by the autogenous vaccine in external replacement gilts (it should be noted that gilts were not vaccinated against *S. suis* before their introduction). Since, these were newly introduced animals, a non-vaccinated control group could be included. Based on the fact that replacement gilts have probably not yet been exposed to circulating *S. suis* strain(s) in the farm, a 3-dose vaccination program was used, as sometimes applied in the field. Nevertheless, the antibody response induced by such a program has never been evaluated. As shown in Fig. 5, total Ig [IgG + IgM] levels against the vaccine strain were already high soon after entry in quarantine (Day − 12) and before vaccination. Indeed, these basal antibodies levels in gilts were similar to those observed in the sows from the first experiment (Fig. 2a). Albeit a significant increase in anti-*S. suis* antibody levels was observed after the 1st vaccine dose and titers continued to increase with successive vaccine doses, they reached highest levels only after the 3rd dose compared to the control group (Fig. 5 and Additional file 1). Interestingly, in the control group, basal levels of antibodies reacting against the vaccine strain also slightly increased over time (Fig. 5 and Additional file 1).

#### Three doses of the autogenous vaccine in gilts increased the maternal antibody transfer to piglets

**Fig. 6:**
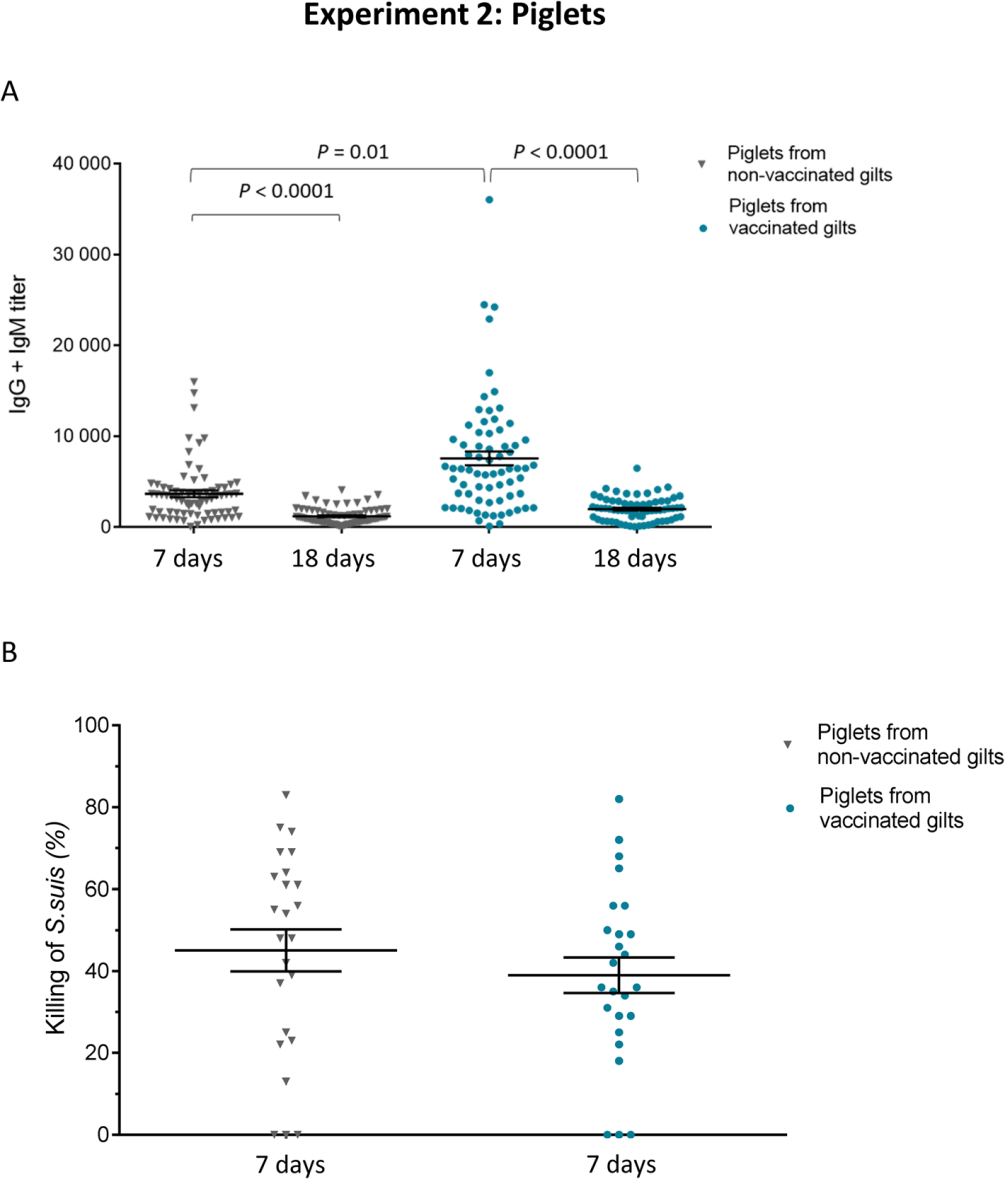
Experiment 2: Kinetics of total Ig against *S. suis* serotype 7 in piglets from either vaccinated or non-vaccinated gilts. **a** Randomly selected 145 piglets (5 piglets/gilt) from vaccinated and non-vaccinated gilts were sampled at 7 and 18 days of age and total Ig [IgG + IgM] titers were determined by ELISA against *S. suis* serotype 7. Individual antibody titers are shown with horizontal bars representing mean ± SEM. Values significantly different are shown in the graph with corresponding *P* value. **b** Blood samples from randomly selected 25 piglets from vaccinated and from non-vaccinated gilts (sampled at 7 days of age) were used to evaluate antibody functionality in an opsonophagocytosis assay (OPA). For OPA, blood leukocytes were mixed with *S. suis* serotype 7 (vaccine strain) at a multiplicity of infection of 0.01. Control sera or sample sera were added to a final concentration of 40% v/v in microtubes which were incubated for 4 h. After incubation, viable bacterial counts were performed, and the percentage of bacterial killing determined. Results are expressed as the % of *S. suis* killing for individual serum, with horizontal bars representing mean ± SEM

The kinetic of total Ig [IgG + IgM] targeting the vaccine strain, induced by a 3-dose vaccination program applied to gilts, was quantitatively evaluated in their piglets at 7 and 18 days of age (Fig. 6a). Antibody levels at 7 days of age were very high in piglets from either vaccinated or non-vaccinated gilts; and these levels were overall variable among individuals. However, piglets from the vaccinated gilts showed significantly higher levels of antibodies than those from the non-vaccinated gilts at 7 days of age (Fig. 6a). As seen in the first experiment, the level of antibodies dropped already at 18 days of age in both groups (Fig. 6a). Vaccination of gilts with a 3-dose vaccination program improved antibody concentration (Fig. 5). Higher maternal antibody concentrations after colostrum uptake should generally lead to a longer period of increased antibody levels; however, this was not observed in piglets from vaccinated gilts at 18 days of age (Fig. 6a). Furthermore, as shown in Fig. 6b, the OPA activity of antibodies was not significantly different in piglets from vaccinated gilts than those from the non-vaccinated gilts at 7 days of age.

#### A 3-dose vaccination program of replacement gilts induced an isotype profile dominated by the IgG1 subclass

**Fig. 7:**
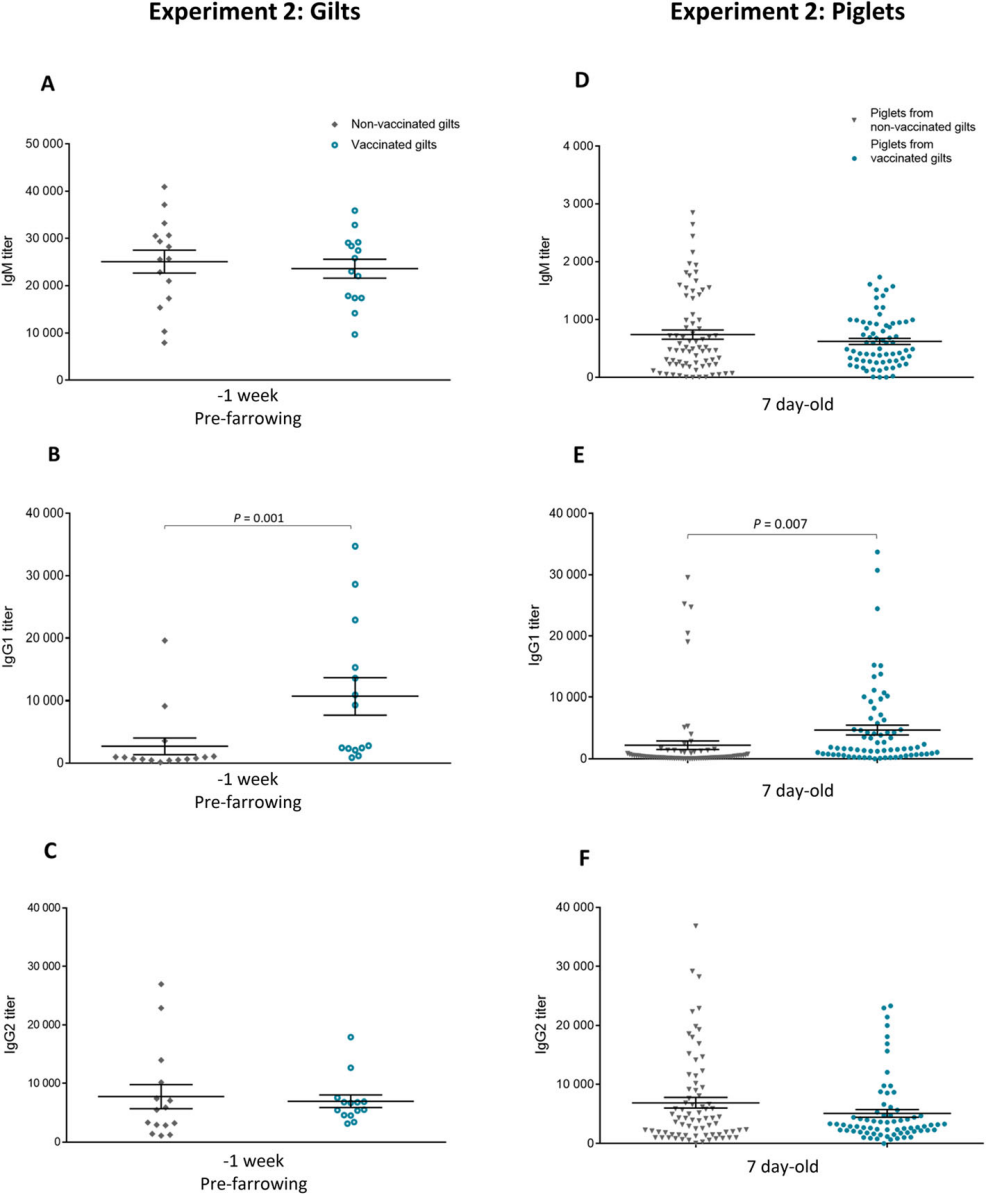
Experiment 2: Isotype profile of antibodies against *S. suis* serotype 7 in vaccinated and non-vaccinated gilts and their piglets. Blood samples were collected 1 week before farrowing from 14 vaccinated and 15 non-vaccinated gilts to analyze the immune response. The vaccination protocol is shown in Fig. 1. IgM (**a**), IgG1 (**b**) and IgG2 (**c**) titers were determined by ELISA. Randomly selected piglets (*n* = 145; 5 piglets/sow) from vaccinated and non-vaccinated gilts were sampled at 7 days of age to evaluate titers of IgM (**d**) IgG1 (**e**) and IgG2 (**f**) against *S. suis* serotype 7. Individual antibody titers are shown with horizontal bars representing mean ± SEM. Values significantly different are shown in the graph with corresponding *P* value

To assess the isotype profile of the vaccine-induced antibody response, serum samples obtained from gilts 1 week pre-farrowing (after 3 vaccinations) and from their piglets at 7 days of age were used to quantify levels of IgM, IgG1 and IgG2 (Fig. 7). The vaccine induced a significant switch to IgG1 in gilts and this profile was also found in piglets (Figs. 7b and e). Indeed, piglets from vaccinated gilts showed a significant increase in IgG1 antibody levels against *S. suis* compared to piglets from non-vaccinated gilts; whereas levels of IgM and IgG2 remained similar between both groups (Fig. 7).

### Experiment 3: recall vaccination at parity 3–4

#### Single-dose boost vaccination pre-farrowing induced a recall IgG1 response

**Fig. 8:**
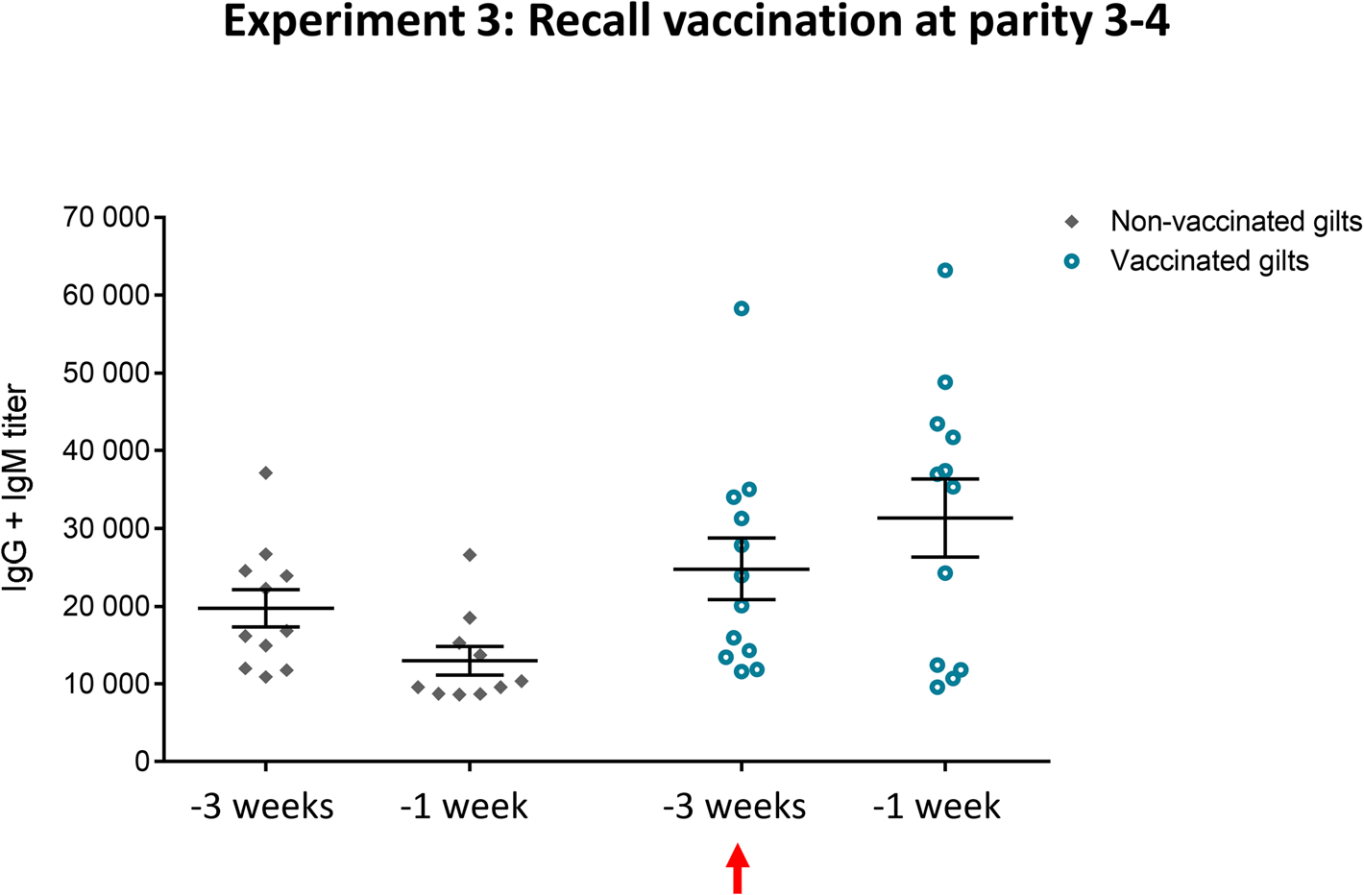
Experiment 3: Kinetics of total Ig against *S. suis* serotype 7 in sows after recall vaccination. Blood samples were collected at 3 weeks and 1 week before farrowing from 12 vaccinated and 11 non-vaccinated gilts (that became sows) from Experiment 2 that received a 4th vaccine dose during their 3rd or 4th parity. The vaccination protocol is shown in Fig. 1. Total Ig [IgG + IgM] titers were determined by ELISA. Individual antibody titers are shown with horizontal bars representing mean ± SEM. Arrow indicates the vaccination dose

**Fig. 9:**
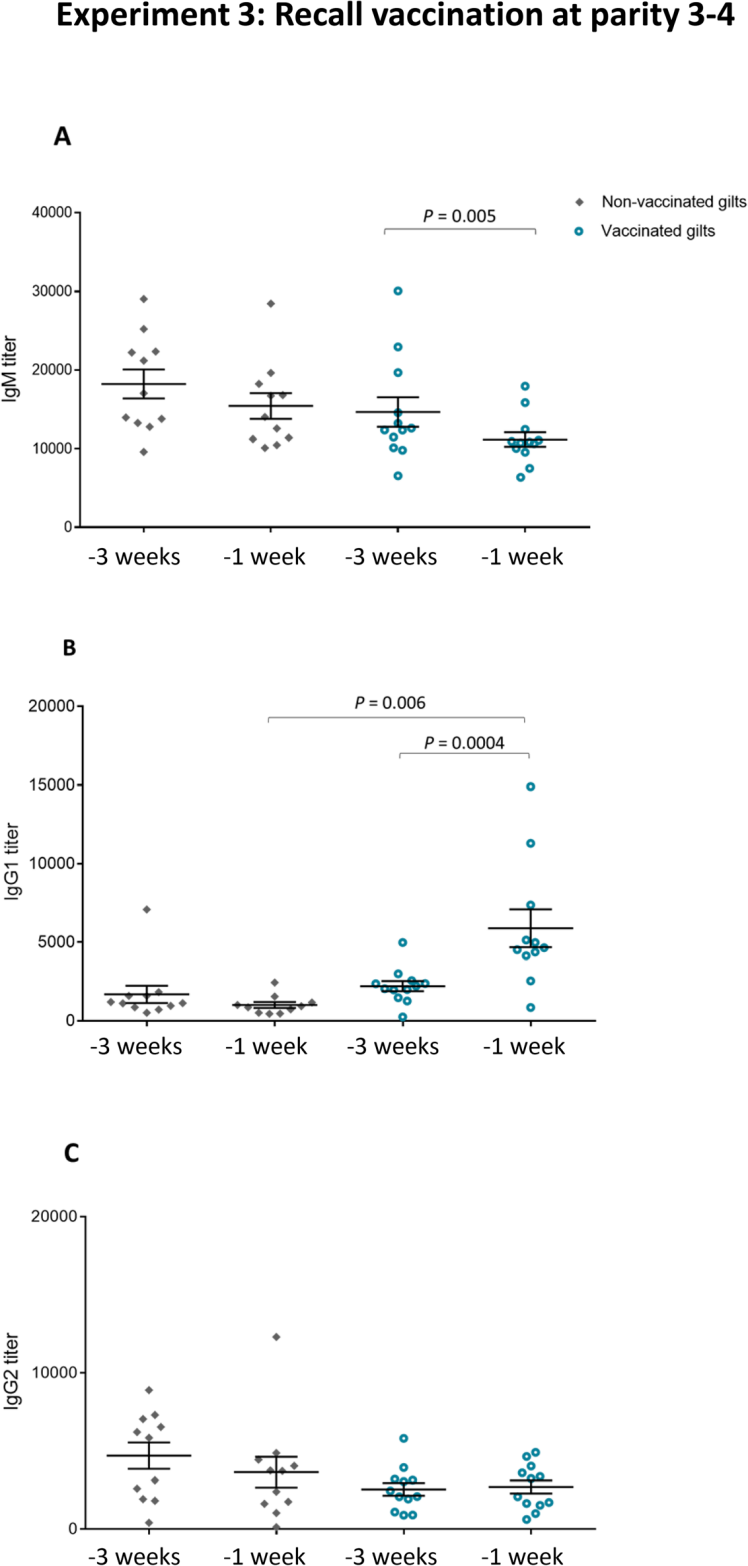
Experiment 3: Isotype profile of antibodies against *S. suis* serotype 7 in sows after recall vaccination. Blood samples were collected at 3 weeks and 1 week before farrowing from 12 vaccinated and 11 non-vaccinated gilts from Experiment 2 that received a 4th vaccine dose prior their 3rd or 4th parity. The vaccination protocol is shown in Fig. 1. IgM (**a**), IgG1 (**b**) and IgG2 (**c**) titers were determined by ELISA. Individual antibody titers are shown with horizontal bars representing mean ± SEM. Values significantly different are shown in the graph with corresponding *P* value

A common practice in farms is to give a single-dose boost vaccination pre-farrowing of previously immunized sows (after their initial 2-dose program) or gilts (after their initial 3-dose program) at each subsequent parity. To assess if this practice provides a recall antibody response, animals from the 2nd experiment received a boost of the same autogenous vaccine at their 3rd or 4th parity depending on their reproductive performance following the first parity. Quantitative antibody responses were analyzed by ELISA before boost-vaccination (3 weeks before parturition) and after vaccination (1 week before parturition) to assess the production of antibodies induced by the vaccine. As observed in previous experiments, basal total Ig [IgG + IgM] antibodies against the vaccine strain were already high prior to vaccination (Fig. 8). Levels of total Ig [IgG + IgM] slightly increased in the vaccinated group after the boost vaccination; yet they did not reach statistical significance compared to the control group (Fig. 8). Nevertheless, analysis of total Ig antibodies might not accurately discriminate differences in individual Ig isotypes. Indeed, an isotype switching from IgM to IgG was clearly observed in the vaccinated group with a significant decrease in IgM (Fig. 9a) and a significant increase of IgG1 (Fig. 9b). This increase of IgG1 after a single-dose boost vaccination is in line with results from the previous experiments (Figs. 3 and 7).

## Discussion

*S. suis* is one of the most important bacterial pathogens in pigs responsible for major economic losses to the swine industry worldwide. Several approaches have been used to develop a vaccine against *S. suis*; however, none has led to an efficient vaccine so far [9]. Besides a commercial bacterin with limited geographical distribution, autogenous bacterins are the only vaccines used to prevent *S. suis* disease [9, 11, 13]. However, scientific studies regarding their ability to confer protection are missing. To the best of our knowledge, only three field studies are available on the efficacy of this preventive approach using autogenous bacterins manufactured by licensed companies [14–16]. Moreover, it is crucial to highlight that autogenous bacterin manufacturing differs from laboratory-made bacterins used in experimental studies. Indeed, several works have evaluated the immunogenicity or protective capacity of experimental bacterins under controlled vaccination/challenge studies [9, 11, 13], but only very few have analyzed the immunogenicity and/or clinical protection using licensed autogenous vaccines under field conditions [14–16]. Overall, results from these studies are controversial, highlighting the importance for more research in this area [11].

Immunization of pre-parturient sows might elicit protective passive maternal immunity in the progeny. In addition, sow vaccination is less costly and labor intensive, thus representing an economical alternative to piglet vaccination [9]. Nevertheless, information on the capacity of this approach to induce passive immunity in piglets is missing. The present field study evaluated a sow vaccination program in a farm with recurrent clinical problems with *S. suis* serotype 7 using an autogenous vaccine manufactured by a licensed company. This study brought together several objectives. One of them was to evaluate the immunological (humoral) response (quantitative and qualitative) induced by the vaccine in sows and gilts. Another objective was to evaluate the vaccine capacity to induce a recall response pre-farrowing. The vaccination protocols used were those currently applied in the farm (thus representing common practice in the field). Finally, the evaluation of maternal immunity transfer to the progeny was an important aspect addressed in this study.

The first observation brought by this study was the relatively high basal levels of antibodies reacting against the vaccine strain. Unexpectedly, these basal levels were similar in replacement gilts and sows. It may be possible that incoming gilts were already exposed to *S. suis* serotype 7 in their farm of origin. However, this observation might also suggest that these antibodies are probably cross-reactive and originated from the normal animal microflora, which includes different serotypes/strains of *S. suis* as well as other streptococci. It should be noted that animals become colonized with *S. suis* very early in life or even during farrowing [1]. Interestingly, the slight increase of basal levels of antibodies reacting against the vaccine strain in control non-vaccinated gilts might reflect the natural gradual exposition of animals to *S. suis*. Similar results were observed in a field study aimed to follow the serological profile of sows and piglets. Sows presented high levels of *S. suis*-reactive antibodies and, in piglets these antibodies naturally increased to reach levels similar to those in sows at 8 weeks of age [6]. These observations confirm the immunological response of animals due to natural exposition. Altogether, these facts highlight the importance of careful analysis of the antibody response induced by the vaccine vs. natural immunity and the importance of including control non-vaccinated groups (when possible) in field studies. This limitation occurred in Experiment 1; nevertheless, the shift towards an IgG1 response may indicate a vaccine effect in sows after a 2-dose vaccination program pre-farrowing. These antibodies (either due to natural exposition and/or vaccination) were highly opsonic, and thus able to eliminate *S. suis* in an in vitro test. Similar findings were observed in a recent field study using autogenous vaccination of sows from a herd that was also experiencing *S. suis* serotype 7 clinical problems [16].

The second observation was that, albeit a rapid vaccine response obtained in replacement gilts after the first vaccine dose, a 3-dose program was nevertheless required to reach a significant increase in antibody levels. These results provide for the first time a scientific justification for implementing such a program in external replacement gilts entering quarantine. This increase in vaccinated-gilt antibody levels translated to a higher maternal immunity present in their piglets compared to those from non-vaccinated gilts. In a previous field study, sows from internal replacement, which have received a 2-dose vaccination program with an autogenous bacterin at their parity 1, also showed increased levels of antibodies. Nevertheless, their piglets presented similar levels of antibodies than piglets from non-vaccinated sows and no clinical protection was observed [16]. These discrepancies might be explained by several variables, including the vaccine formulation, the use of 3 doses vs. 2 doses, and internal vs. external replacement sources among other herd-specific factors. In spite of these differences, a common feature observed between the two field studies was that duration of maternal immunity drops very fast independently of the vaccination program. This drop in maternal immunity occurs at the moment of high vulnerability of weaned piglets to *S. suis* infection [1].

Information regarding maternal immunity is limited: only a few experimental studies in sows using laboratory-made bacterins were reported and their results are contradictory. Administration of a *S. suis* serotype 14 bacterin to sows reduced some *S. suis* related clinical signs but not mortality in their piglets [17]. Unfortunately, antibody levels were not measured in that study. Another study has shown that sow vaccination with a *S. suis* serotype 2 bacterin results in serum antibody titers significantly higher in vaccinated sows and in their suckling piglets. However, these maternal antibodies declined in the following weeks and clinical protection was not observed at 8 weeks of age [5]. In the study by Blouin et al., sow vaccination with a *S. suis* serotype 2 bacterin resulted in a poor increase of antibody titers and low transfer of maternal immunity to the litters [10]. Finally, sow immunization with *S. suis* serotype 2 bacterin plus recombinant “surface antigen one” (Sao) protein resulted in increased levels of specific antibodies, against the whole bacteria and Sao, in piglets. Interestingly, the serum titer against whole bacteria, but not Sao, was remarkably reduced in piglets at 6 week-old [18].

Albeit hard to compare due to clear experimental differences, altogether these studies (either field or experimental) suggest the need of optimization of the vaccination program and/or the vaccine formulation in order to induce lasting maternal immunity in piglets. The 3-dose vaccination program of gilts herein described used an autogenous vaccine formulated with Alhydrogel™, whereas the 2-dose vaccination program of sows in the previous field study used an autogenous vaccine prepared with an oil-in-water emulsion (confidential formulation) [16]. Aluminum salts are generally considered to be weaker adjuvants than emulsion adjuvants; yet, when using this adjuvant in Experiment 2, a significant increase in maternal immunity transfer to piglets was observed. These observations imply that multiple doses might overcome, at least in part, the limited immunogenicity of some autogenous vaccine formulations. Bacterin hyperimmunization is an old, still successful, approach to induce high levels of antibodies that was reported in the 90s [12]. The aforementioned experimental sow vaccination studies either used water-in-oil-in water (w/o/w) adjuvant [18], Emulsigen® [5] or an oil-in-water emulsion [17]. These divergent formulations, including the addition of a recombinant protein in the study by Hsueh et al. [18], might explain differences in antibody levels, antibody quality (see below), duration of maternal immunity in piglets and, when evaluated, protective capacity. In a vaccination (challenge) study in piglets under experimental conditions, protection was only recorded when using a bacterin formulated with a water-in-oil emulsion compared to a bacterin adjuvanted with Alhydrogel™ [19]. Comparative studies in sows are nevertheless not available.

Indeed the adjuvant used in the formulation can markedly influence not only the quantity (titers) but also the quality (isotype) of the antibody response induced by the vaccine [20, 21]. The right choice of adjuvant can also contribute to reducing the number of vaccine doses required [22]. In our study, the vaccine formulation with Alhydrogel™ induced a clear switch to IgG1 in sows and gilts and, consequently predominated in piglets born from vaccinated gilts. In the previous field study, the autogenous vaccine prepared with an oil-in-water emulsion induced a switch to either IgG1 or both IgG1 and IgG2 depending on the bacterial serotype included in the vaccine [16]. Similarly, in the experimental sow vaccination study using a bacterin adjuvanted with Emulsigen®, both anti-MRP IgG1 and IgG2 were observed in sow-derived sera and colostrum [5]. The isotype profile is known to correlate with the capacity of the antibody to induce opsonophagocytosis [21, 23]. This mechanism is key for *S. suis* elimination, since this encapsulated extracellular bacterium is highly resistance to phagocytosis in the absence of opsonizing antibodies [24–28]. Importantly, levels of opsonizing antibodies were found to correlate with the protection elicited by bacterin vaccination against *S. suis* [29]. Nevertheless, the functionality of the different IgG subclasses in the swine species remains to be fully elucidated [30]. Also, in aforementioned studies, IgG subclasses were defined based on reagents used for their detection and do not represent the complexity of swine IgG subclasses as reported by transcriptome analysis [30].

The target antigen must also be taken into the equation in order to determine the effectiveness of a given vaccine formulation [5, 16, 20, 31]. It should be noted that independently of the vaccination program (and the induced isotypes) high levels of opsonic antibodies are detected in sows, as reported herein and in previous studies [5, 16]. However, this functionality falls quickly in piglets at 3 weeks of age [5, 16]. Furthermore, in experiment 2, the capacity of vaccine-induced antibodies to eliminate *S. suis* in an in vitro test was not improved in spite of higher levels of maternal antibodies in 7-day-old piglets from vaccinated gilts. This might suggest that levels and functionality do not necessarily correlate; at least under the conditions evaluated herein. Therefore, more research is needed on the link between isotypes, opsonic activity, and protection of weaned piglets after applying an autogenous vaccine to sows. Unfortunately, a clinical follow-up at the nursery (in terms of mortality or morbidity) was not possible during this study; this is a limitation precluding evaluation of efficacy of the vaccination approach used in the farrow-to-wean farm.

Finally, we assessed the duration of the vaccine-induced immunity by injecting a “boost” vaccine dose to gilts (that became sows) from Experiment 2 at their 3rd or 4th parity. This is another common practice in the field but no scientific data are available to support this prevention strategy. Albeit the boost vaccination failed to increase total Ig levels in vaccinated sows, a clear recall effect (memory response) to the vaccine was evidenced by an isotype switching from IgM to IgG1 against the *S. suis* vaccine strain. This observation suggests that a recall response can be induced by a single vaccine boost pre-farrowing after the initiation of the vaccination program in the herd. This fact further implies the induction of a memory response by the vaccine. Yet, the protective effect on the progeny remains to be elucidated.

## Conclusion

In summary, a 2-dose vaccination program of sows and a 3-dose vaccination program of replacement gilts both induced an increase in antibody titers and isotype switch pre-farrowing. The 3-dose program also resulted in increased passive immunity transfer to piglets, mainly of the Ig isotype induced by the vaccine. This strategy seems to favor the establishment of an immunological memory in sows. Nevertheless, duration of maternal immunity in weaned piglets remains a concern and a challenge that might be overcome by improvement of the vaccine formulation. Indeed, this promising approach requires extensive and comparative scientifically sound studies to evaluate the most efficacious way to prepare the vaccine, the adjuvant to be included, the number of doses, the real benefit of vaccinating sows, or piglets or both. Finally, it is important to remember that the overall efficacy of autogenous vaccines cannot be determined based on results obtained with one particular batch of vaccine prepared by a single licensed laboratory. Methods used for the vaccine production, bacterial concentration and the adjuvant used (among other variables) may highly influence the results obtained [11].

## Methods

### Pig herd and vaccine preparation

A farrow-to-wean farm located in the Province of Quebec, Canada, was selected for this study. At the time of the study, clinical problems occurred after weaning (once the piglets arrived to the nursery site). This maternity was the only source of piglets in that nursery. Piglets presented a history of clinical problems caused by *S. suis* (mainly arthritis and meningitis, as confirmed by necropsy and bacteriological diagnostic). Of a total of seven isolates (from different piglets), four were serotyped and all were serotype 7. At the initiation of the *S. suis* vaccination program, a new source of replacement gilts was introduced to the farrow-to-wean farm.

The autogenous vaccine was prepared by a private company possessing the official license for manufacturing such products (i.e. authorized by the local authorities to produce the vaccine). It was composed by *S. suis* serotype 7 strain 1750775 (isolated from a diseased piglet in the farm), combined with a strain of *Staphylococcus hyicus* and a strain of *Actinobacillus suis* and adjuvanted with Alhydrogel™ (aluminum hydroxide adjuvant). Bacteria were formalin-killed. *S. suis* serotype 7 strain 1750775 was confirmed by serotyping (PCR and coagglutination test) by the Diagnostic Service of the Faculty of Veterinary Medicine (University of Montreal). The farm had previous porcine reproductive and respiratory syndrome virus (PRRSV) disease episodes; however, at the moment of the study, the farm was considered provisional negative [32]. The regular vaccination schedule at the farm for gilts was as follow: Flusure XP/Farrowsure Gold® (swine influenza virus, parvovirus, leptospirosis, and erysipelas) and Circumvent® PCV-M G2 (porcine circovirus type 2 and *Mycoplasma hyopneumoniae*). For sows, the vaccination program of the farm included Prosystem® RCE (rotavirus, *Escherichia coli* and *Clostridium perfringens* type C) and Farrowsure Gold® vaccine (parvovirus, leptospirosis, and erysipelas). No ethical statement was required for this study as the protocol used was part of normal interventions in the farm and performed by the veterinarian in charge, as stated by the Animal Welfare Committee of the University of Montreal. The study ended at weaning and animals were transferred to the nursery as per the normal procedures in commercial farms.

### Vaccine protocols

When the study started, the *S. suis* vaccination program was already in progress. Thus, for **Experiment 1** (Fig. 1; Experiment 1-A), 25 pre-parturient sows (never vaccinated against *S. suis* before) at different parities and in the same gestation stage were selected. Negative controls (i.e. non-vaccinated sows) could not be included. Sows received 2 doses of 2 ml of the autogenous vaccine by intramuscular injection. The first vaccination was performed at 7 weeks before parturition and the second vaccination was performed 3 weeks pre-farrowing. To evaluate transfer of maternal immunity, 5 piglets per vaccinated sow (*n* = 125) were randomly selected (Fig. 1; Experiment 1-B).

In **Experiment 2**, 29 replacement gilts at parity 0 were divided into a vaccinated group (*n* = 14) and a non-vaccinated group (*n =* 15). The vaccinated group received 3 doses of 2 ml of the same autogenous vaccine by intramuscular injection. The two first vaccinations were performed during quarantine at 21 day-interval. The third (boost) vaccination was performed during gestation at 3 weeks before farrowing (Fig. 1; Experiment 2-A). To evaluate transfer of maternal immunity, 5 piglets per gilt (*n* = 145) were randomly selected (Fig. 1; Experiment 2-B).

In **Experiment 3**, 23 animals from experiment 2-A were followed at their 3rd or 4th parity depending on their reproductive performance following the first parity; 12 came from the vaccinated group and 11 came from the control non-vaccinated group (unfortunately samples were not available for the 2nd parity). The vaccinated group received a 4th dose of vaccine (boost) at 3 weeks before farrowing (Fig. 1, Experiment 3).

### Blood sampling

For Experiment 1, blood samples from sows were collected prior to vaccination, after the first and after the second vaccination (Fig. 1; Experiment 1-A). Blood samples for randomly selected (and tagged) piglets (*n =* 125) from the vaccinated sows were collected at 7 and 18 day-old (Fig. 1; Experiment 1-B). For Experiment 2, blood samples were collected from vaccinated and non-vaccinated gilts. The two first samples were collected during quarantine (before and after the primo vaccination). The 3rd blood sample was during gestation (after the second vaccination); and the fourth blood sample was taken after the third vaccine dose (i.e. 1 week before farrowing) (Fig. 1; Experiment 2-A). Blood samples for randomly selected (and tagged) piglets (*n =* 145) were collected at 7 and 18 day-old (Fig. 1; Experiment 2-AB). For Experiment 3, blood samples were collected prior to vaccination and 1 week before farrowing (Fig. 1, Experiment 3). Serum samples were stored at − 20 °C until used to evaluate the immune response by ELISA and by OPA assay (described below).

### Enzyme-linked immunosorbent assay (ELISA) for pig immunoglobulin (Ig) titers

The *S. suis* strain used in the autogenous vaccine was also used as the coating for ELISA Polysorb plates (Nunc-Immuno; Thermo Scientific, Mississauga, ON, Canada). The ELISA protocol was adapted from Corsaut et al. [16]. Briefly, bacteria were grown overnight onto 5% sheep blood agar plates at 37 °C, and isolated colonies were cultured in 5 ml of Todd-Hewitt broth (THB) (Becton Dickinson, Mississauga, ON, Canada) for 8 h at 37 °C with agitation at 120 rpm. Then, 10 μl of 1/1000 dilution of 8-h cultures were transferred into 30 ml of THB and incubated for 16 h at 37 °C with agitation. Stationary-phase bacteria were washed in phosphate-buffered saline (PBS) at pH 7.3. Bacteria pellet was then adjusted to a concentration equivalent to 10^7^ CFU/ml. Plates were coated with 100 μl/well with the whole bacteria suspension, air-dried during 2 days at room-temperature (RT), and finally fixed with 50 μl/well of 100% methanol. After evaporation of methanol, plates were stored at RT until use. After washing, 100 μl of serial 2-fold based dilutions of pig sera (in PBS containing 0.05% (v/v) Tween 20) were added to each well and incubated for 1 h at RT. For titration of porcine total Ig [IgG + IgM] or IgM, plates were incubated with peroxidase-conjugated goat anti-pig total Ig [IgG + IgM] (Jackson ImmunoResearch, West Grove, PA) or IgM (AbD Serotec, Raleigh, NC) antibodies for 1 h at RT. For porcine IgG1 or IgG2 detection, mouse anti-porcine IgG1 or IgG2 (BioRad, Mississauga, ON, Canada) was added for 1 h at RT. After washing, peroxidase-conjugated goat anti-mouse IgG (Jackson ImmunoResearch) was added for 1 h at RT. Plates were developed with 3,3,5,5-tetramethylbenzidine (TMB; InvitroGen, Burlington, ON, Canada) substrate, and the enzyme reaction was stopped by addition of 0.5 M H_2_SO_4_. Absorbance was read at 450 nm with an ELISA plate reader. The reciprocal of the last serum dilution that resulted in an optical density at 450 nm (OD_450_) of ≤0.2 (cutoff) was considered the titer of that serum. To control inter-plate variations, an internal reference positive control was added to each plate. This positive control was composed by a pool of serum of six sows randomly selected in the farm that showed high ELISA values against *S. suis* serotype 7 because of their natural exposition to this serotype in the farm. Reaction in TMB was stopped when an OD_450_ of 1.0 was obtained for the positive internal control. Optimal dilutions of the positive internal control sera and anti-porcine antibodies or conjugates were determined during preliminary standardizations.

### Opsonophagocytosis assay

The OPA test was performed as previously published [16]. Briefly, whole blood, as a source of total phagocytic cells, was obtained from young naive (not experimentally infected) piglets originating from a farm without clinical problems with *S. suis*. Blood was prepared in complete cell culture medium (RPMI 1640 supplemented with 5% heat-inactivated fetal bovine serum, 10 mM HEPES, 2 mM L-glutamine, and 50 μM 2-mercaptoethanol) to obtain 3 × 10^7^ leukocytes/ml. All reagents were from Gibco (InvitroGen). All blood preparations were kept at RT. Using washed bacterial cultures grown as described above, final bacterial suspensions were prepared in the same complete cell culture medium to obtain a concentration of 6 × 10^5^ CFU/ml. The number of CFU/ml in the final suspension was determined by plating samples onto THB agar (THA). All bacterial suspensions were kept on ice. Diluted whole blood at 2.5 × 10^6^ leukocytes was mixed with 2.5 × 10^4^ CFU of *S. suis* type 7 strain 1750775 to obtain a multiplicity of infection [MOI] of 0.01. Control and sample sera from immunized animals were added to a concentration of 40% v/v in microtubes to a final volume of 200 μl. Control sera came from naïve pigs (absorbed against *S. suis* serotype 7 and presenting negative ELISA values), and positive sera were obtained and pooled from sows (originated from the same farm and presenting high ELISA values). The tube tops were pierced using a sterile needle and were incubated for 4 h at 37 °C with 5% CO_2_, with gentle agitation. After incubation, viable bacterial counts were performed on THA using an Autoplate 4000 automated spiral plater. The percentage of bacterial killing was determined using the following formula:

% Bacteria killed = [1 - (Bacteria recovered from sample tubes / Bacteria recovered from negative control tube with control serum)] × 100.

### Statistical analyses

Titer data were log-10 transformed to normalize the distributions. Linear mixed models of different structures were used to analyze the data. When an overall significant effect was detected, priori contrasts were performed to examine differences between pairs of means adjusting the alpha threshold downward with the Benjamini-Hochberg sequential procedure.

The structure of the linear mixed models varied depending on the study factors. For sows in Experiment 1, bleeding (before and after vaccine doses) was the within-subject factor. For piglets in Experiment 1, bleeding (at 7 and at 14 days of age) was the within-subject factor and sow identification (id) was a random effect. For gilts in Experiment 2, bleeding (1 to 4) was the within-subject factor and group (vaccinated or not) was the between-subject factor. To compare IgG1 and IgG2 between gilts at the last bleeding, a linear model was used with group as between-subject factor. For piglets in Experiment 2, bleeding (at 7 and 18 days of age) was the within-subject factor, group (sow vaccinated or not) was the between-subject factor and sow id was a random effect. To compare IgG1 and IgG2 between piglets of the two groups at the first bleeding, group (sow vaccinated or not) was the between-subject factor and sow id was a random effect. For gilts in Experiment 3, bleeding (at 3 weeks and 1 week pre-farrowing) was the within-subject factor and group (vaccinated or not) was the between-subject factor. Statistical analyses were carried out in SAS v.9.4 (Cary, N.C.). The level of statistical significance was set at 0.05.

## Supplementary Information

**Additional file 1.**

## Data Availability

The materials and data not presented in this manuscript are available from the corresponding author upon request.

## Abbreviations

PRRSV: Porcine Reproductive and Respiratory Syndrome Virus
Sao: Surface antigen one
RT: Room temperature
PBS: Phosphate-buffered saline
THB: Todd-Hewitt broth
THA: Todd-Hewitt broth agar
MOI: Multiplicity of infection
CFU: Colony forming unit
TMB: 3,3,5,5-tetramethylbenzidine
OPA: Opsonophagocytosis assay
