# Supplementary material for: Immunogenicity study of a Streptococcus suis autogenous vaccine in preparturient sows and evaluation of passive maternal immunity in piglets
Source: BMC Vet Res. 2021 Feb 5;17:72. doi: 10.1186/s12917-021-02774-4 (PMC7866767; doi:10.1186/s12917-021-02774-4)
Supplement: Supplementary file 1 — Additional file 1. [file 12917_2021_2774_MOESM1_ESM.pdf]

Additional file 1

Immunogenicity study of a *Streptococcus suis* autogenous vaccine in preparturient sows and evaluation of passive maternal immunity in piglets

Lorelei Corsaut[1], Léa Martelet[1], Guillaume Goyette-Desjardins[1], Guy Beauchamp[2], Martine Denicourt[1], Marcelo Gottschalk[1] and Mariela Segura[1*]

[1]Research Group on Infectious Diseases in Production Animals (GREMIP) and Swine and Poultry Infectious Diseases Research Centre (CRIPA), Faculty of Veterinary Medicine, University of Montreal, 3200 Sicotte St., Saint-Hyacinthe, Quebec J2S 2M2, Canada

[2]Biostatistics Office, Faculty of Veterinary Medicine, University of Montreal, Saint-Hyacinthe, Quebec, Canada

[*]Correspondence: mariela.segura@umontreal.ca

Experiment 2: Gilts

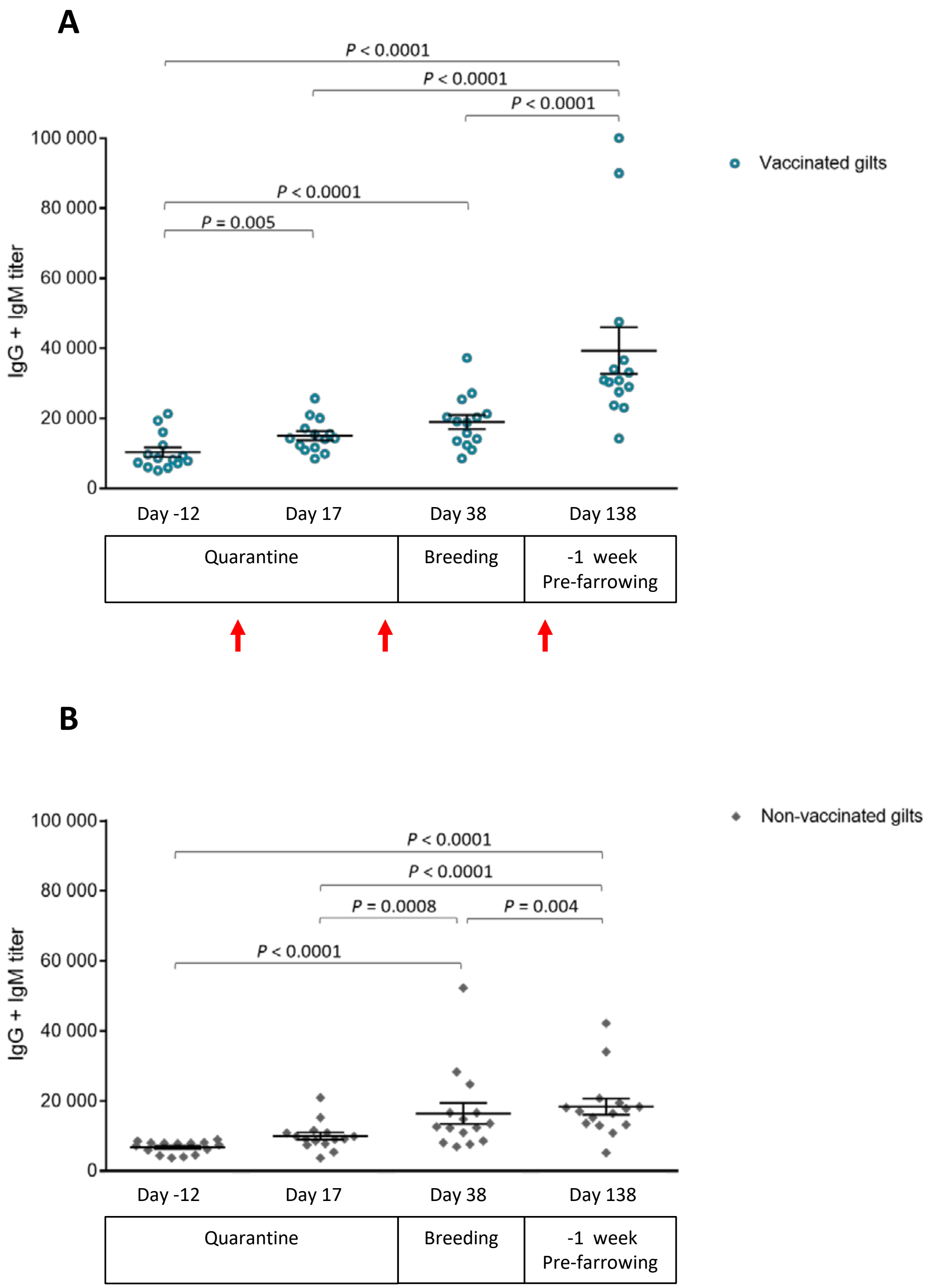

Additional file 1 legend:

Experiment 2: Kinetics of total Ig against *S. suis* serotype 7 from either vaccinated gilts (A) or non-vaccinated gilts (B). Non-vaccinated and vaccinated gilts from **Figure 5** are shown here in 2 separated graphics **(A and B)** to better display all the statistical analyses performed**.** Blood samples were collected from 14 vaccinated **(A)** and 15 non-vaccinated gilts **(B)** prior to vaccination at day-12, at day 17 (2 weeks after the 1st vaccination), at day 38 (2 weeks after the 2nd vaccination) and at day 138 (2 weeks after the 3rd vaccination and corresponding to 1 week pre-farrowing). The vaccination protocol is shown in **Figure 1**. Total Ig [IgG + IgM] titers were determined by ELISA. Individual antibody titers are shown with horizontal bars representing mean ± SEM. Values significantly different are shown in the graph with corresponding *P* value. Arrows indicate 1st, 2nd and 3rd vaccination doses.
